# Supplementary material for: Hnf1b renal expression directed by a distal enhancer responsive to Pax8
Source: Sci Rep. 2022 Nov 19;12:19921. doi: 10.1038/s41598-022-21171-x (PMC9675860; doi:10.1038/s41598-022-21171-x)
Supplement: Supplementary file 1 — Supplementary Information. [file 41598_2022_21171_MOESM1_ESM.pdf]

Figure S1a

Chromatin signature of a genomic region including CNS1 and CNS2 in Homo sapiens. Representative DNase-seq tracks for a 123 kb genomic region in human (chr17: 37,714,024-37,837,224 – GRCh38/hg38 assembly) from ENCODE database (https://www.encodeproject.org) for the following biological materials:

1 : kidney tissue (female embryo 108 days, ENCSR941DTJ)
2 : Epithelial of proximal tubule primary cells (ENCSR000EPW)
3 : Kidney tubule primary cells (female adult 80 years, ENCSR17IWT)
4 : Kidney epithelial primary cells (ENCSR00EOL)
5 : Caco-2 cell line (ENCSR000EMI)
6 : A549 cell line (ENCSR000ELW)
7 : Spinal cord (male embryo 96 days, ENCSR788SOI)
8 : Neural progenitors (female embryo 5 days neural progenitor in vito differentiated cells originated from H9, ENCSR963ALV)

A bottom track shows sequence conservation among the indicated vertebrate species. The observed region partly overlaps with the location of HNF1b transcript as indicated in the last bottom track. The blue box indicates CNS1 and the orange one CNS2. All tracks were visualized using UCSC browser (http://genome.ucsc.edu) and vertical viewing range setting.

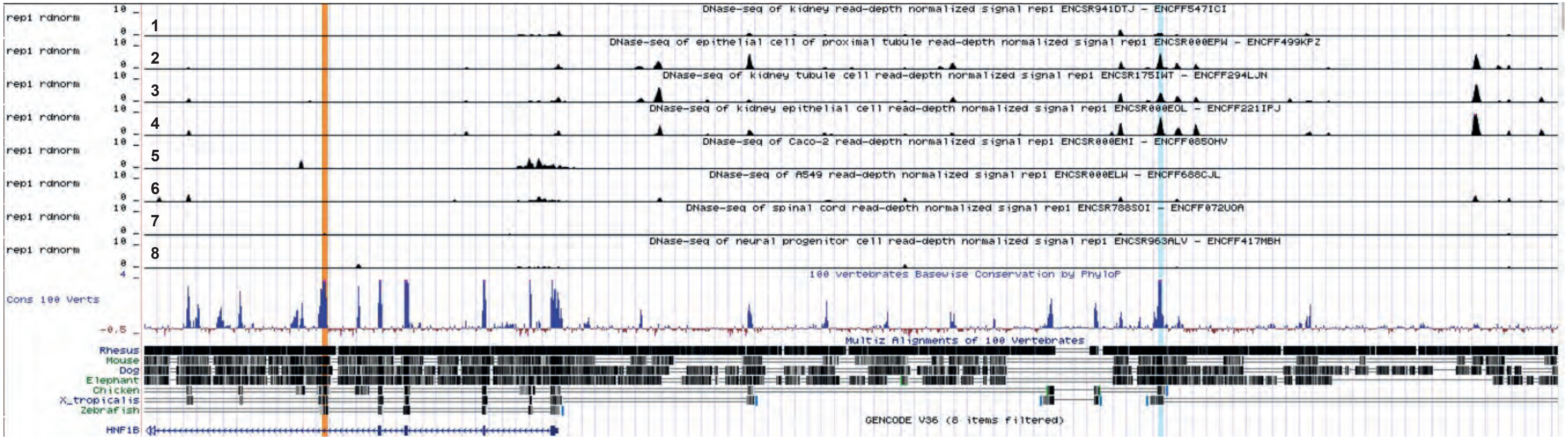

CNS2 in orange

CNS1 in blue

Figure S1b

Chromatin signature of a genomic region including CNS1 and CNS2 in Mus musculus - Representative ATAC-seq and histone modifications tracks as indicated for a 65 kb genomic region in mouse (chr11: 83,810,000-83,875,000 - GRCm38/mm10 assembly) from ENCODE database (https://www.encodeproject.org) in kidney, lung and liver of E15.5 mouse embryo. The blue box indicates CNS1 and the orange one CNS2. All tracks were visualized using UCSC browser (http://genome.ucsc.edu)**and vertical viewing range setting.**

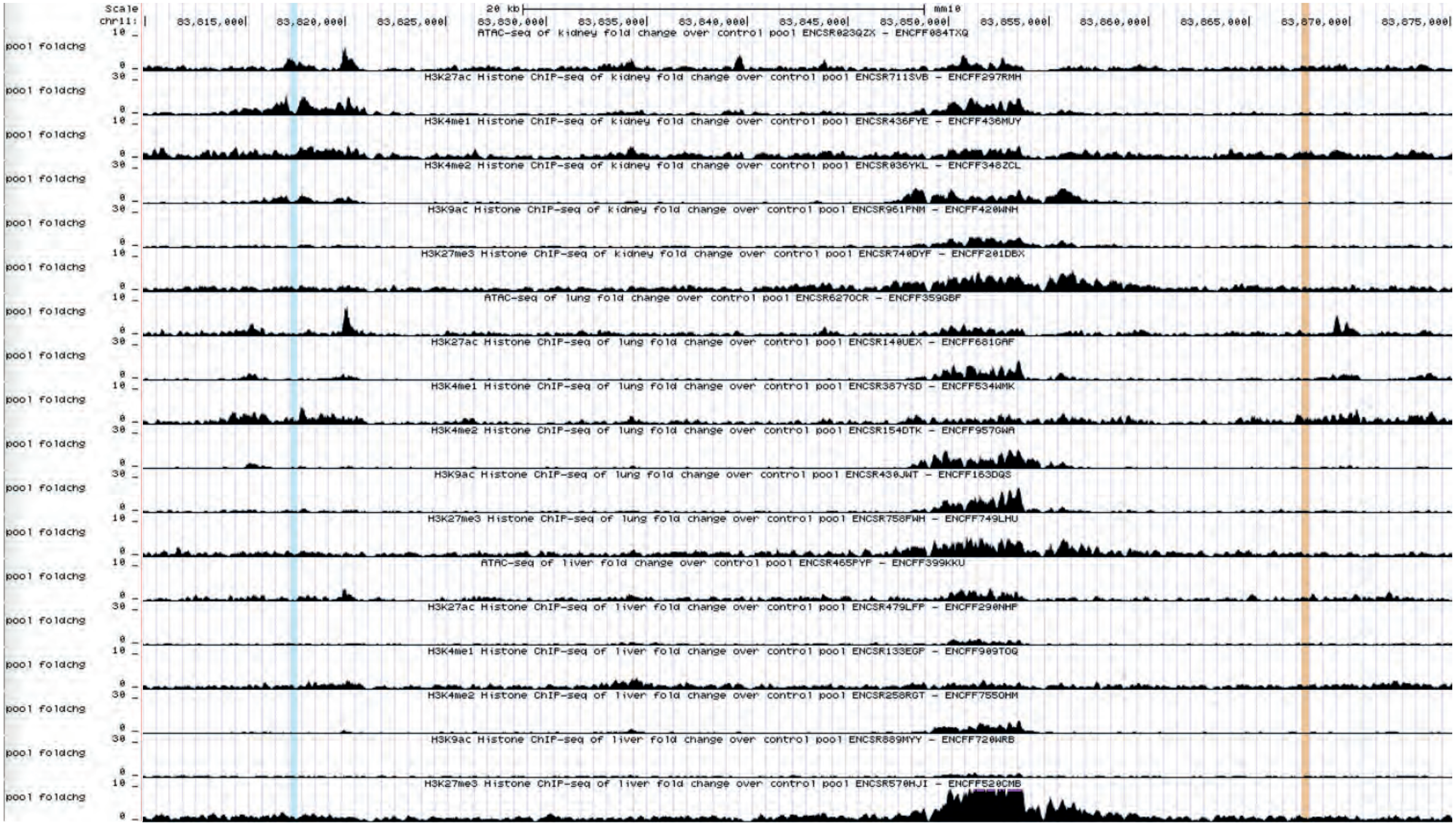

CNS1 in blue **CNS2 in orange**

Figure S1c

Chromatin signature of a genomic region including CNS1 and CNS2 in Xenopus tropicalis. Representative ChiP-seq tracks for the indicated proteins and embryo stages 10.5, 16 and 30 for a 47 kb genomic region in Xenopus tropicalis (chr02: 48,882,893-48,930,436 – xT9_0 Assembly) (http://www.veenstralab.nl/trackhubx.htm, Hontelez S. et al, 2015). The blue box indicates CNS1 and the orange one CNS2.

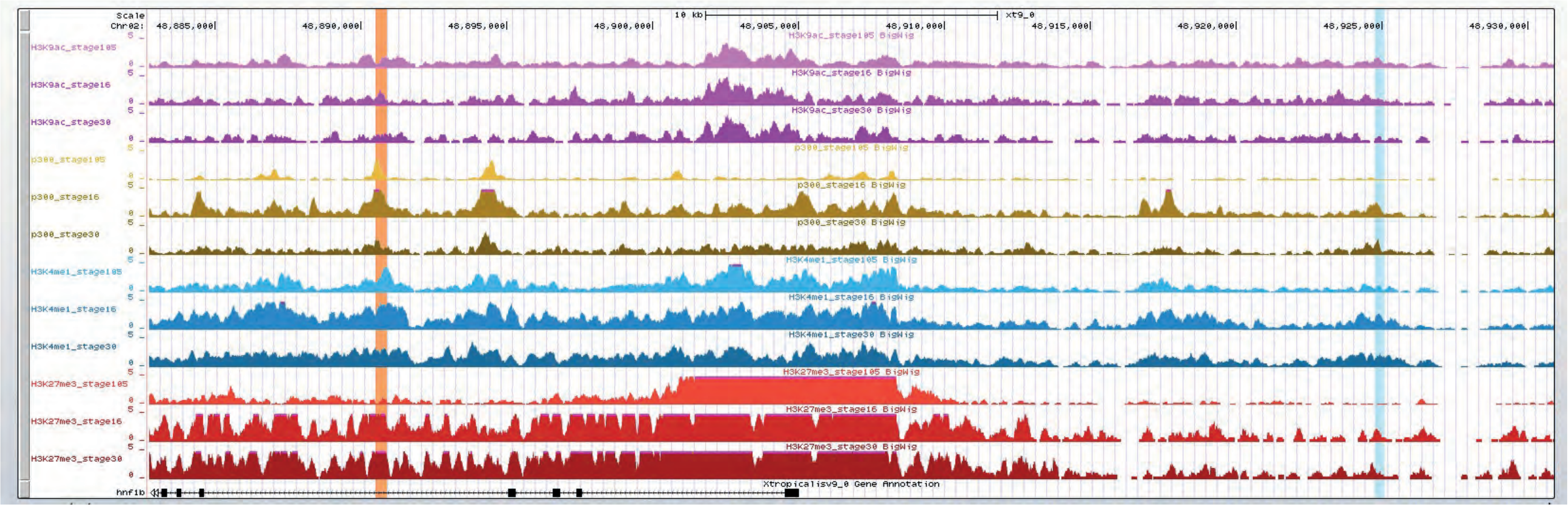

CNS2 in orange **CNS1 in blue**

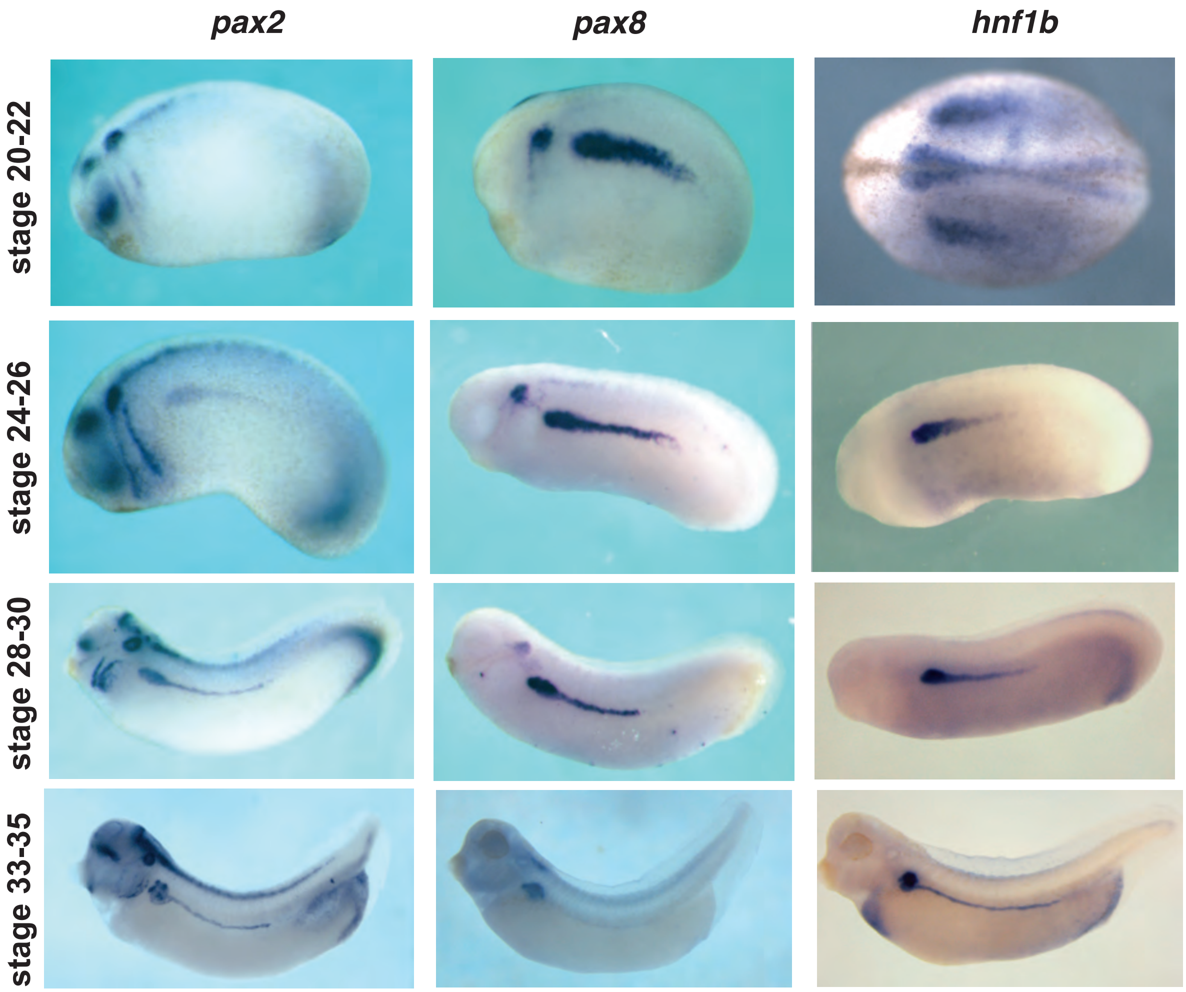

Figure S2

In situ hybridization for *pax2, pax8 and hnf1b* in *Xenopus laevis* embryos at the indicated stages

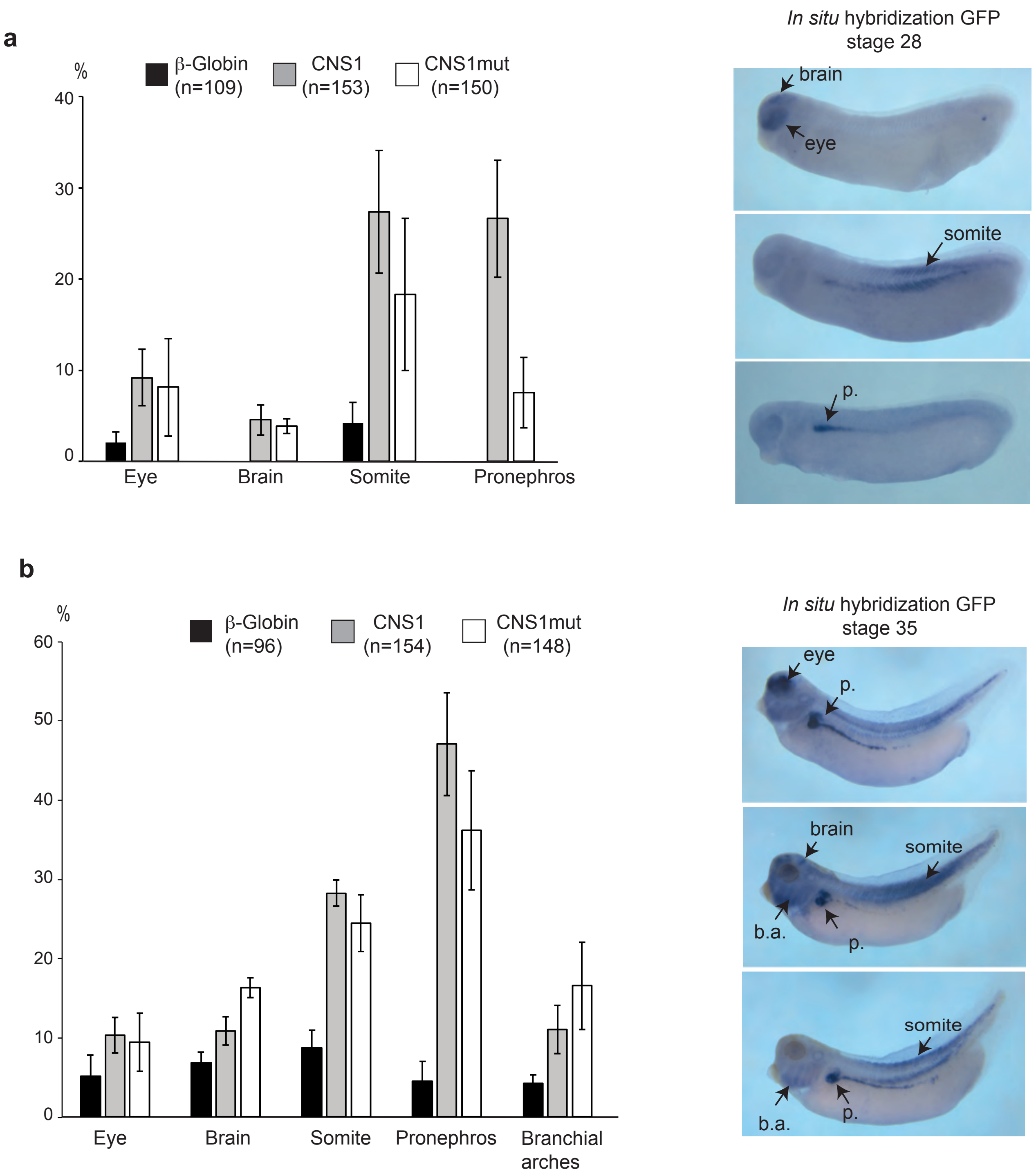

Figure S3

CNS1 activity in Xenopus laevis transgenic embryos generated by the I-SceI meganuclease method. Histograms indicating the percentage of F0 transgenic embryos generated with CNS1 -eGFP, CNS1 mut -eGFP or the control vector -globin basal promoter, showing GFP expression in the indicated tissue and/or organ at stage 28 and stage 35. Results corresponding to three independent experiments. On the right, examples of GFP in situ hybridization performed on CNS1-eGFP transgenic embryos showing expression in the different tissue and organs. b.a.: branchial arches, p: pronephros

-

-

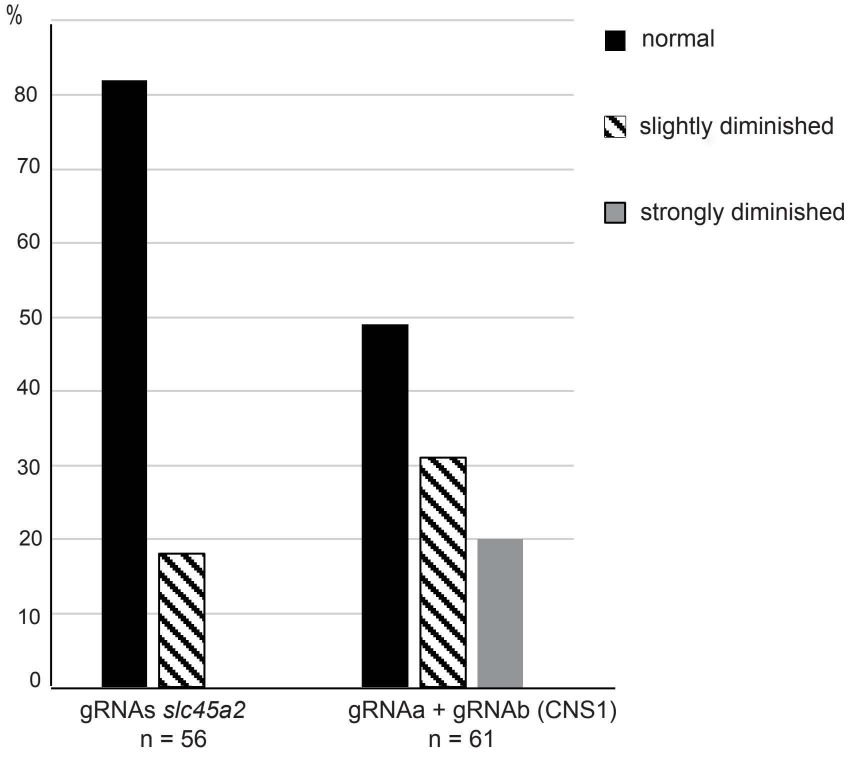

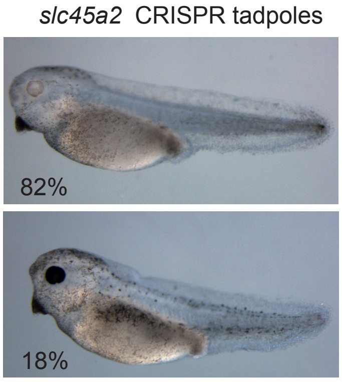

Figure S4

Comparison of CNS1 and slc45a2 editing on hnf1b expression in Xenopus tropicalis embryos. Embryos were injected with Cas9 protein and gRNAs targeting slc45a2 or gRNAs targeting CNS1 (gRNAa-gRNAb). Embryos were raised to stage 28 and analyzed by in situ hybridization for hnf1b and classified into three groups according to their hnf1b pronephric expression (normal, slightly diminished, strongly diminished). The histogram shows the percentage of embryos for each group. n indicates the total number of analyzed embryos. Average values from 2 independent experiments. Fisher's exact test gives a p-value of 3.4 10-5. The efficiency of targeting slc45a2 was checked by analyzing eye and skin pigmentation at tadpole stage. On the right are shown representative embryo at tadpole stage generated with gRNAs targeting slc45a2. 82 % of injected embryos (46/56) exhibit pigment defect.

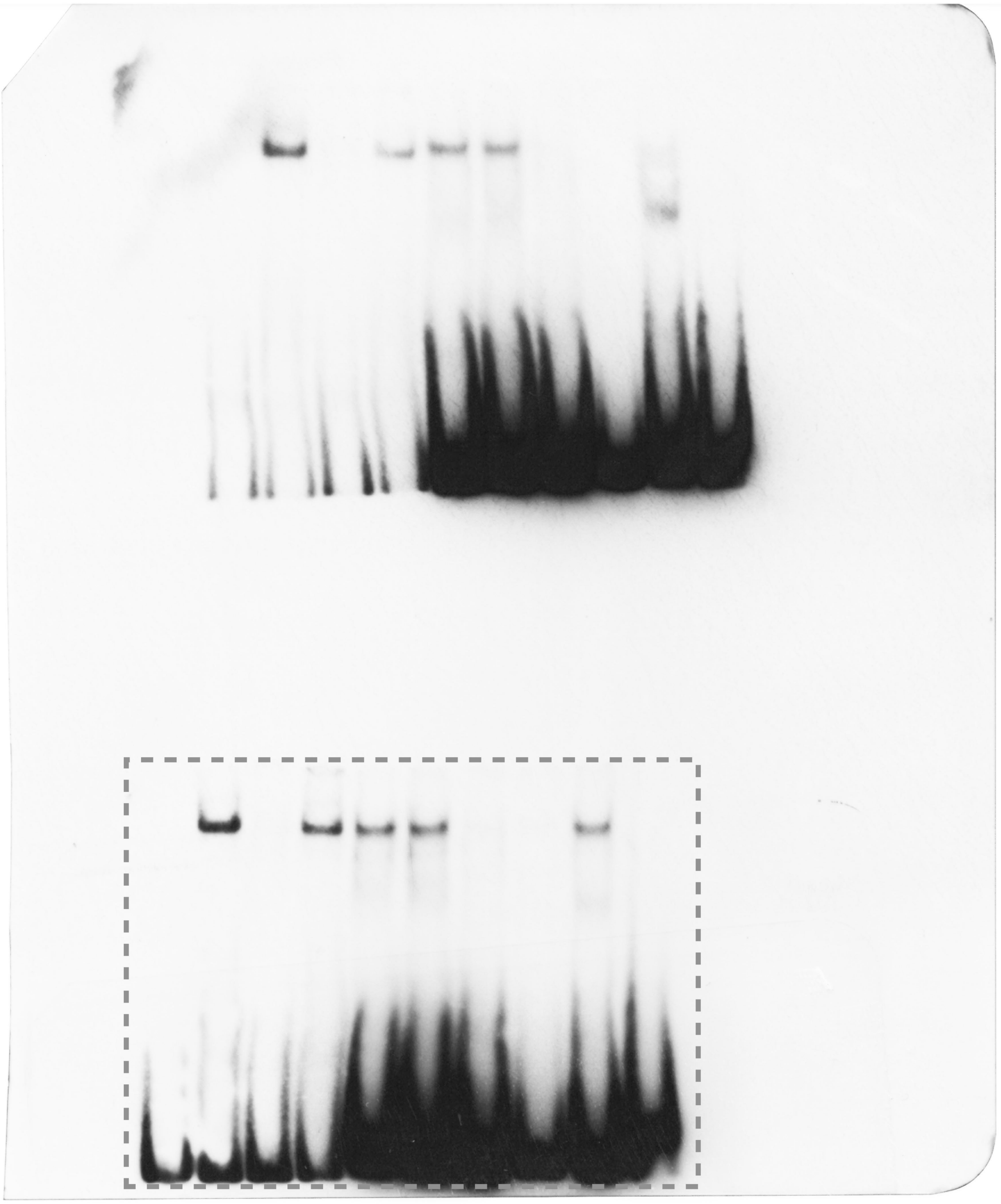

Figure S5

Uncropped autoradioradiography of the whole membrane presented in Figure 1

Supplementary Table 1: *In silico* identification of conserved TFs binding sites conserved in *Mus musculus* and *Xenopus tropicalis* CNS1 using R-vista 2.0 (https://rvista.dcode.org).

Conserved TF binding site	strand	Location (Mus musculus)	Sequence (Mus musculus)	Location (Xenopus tropicalis)	Sequence (Xenopus tropicalis)	Matrix score
V$RBPJK_01	+	23-33	aaTGTGTGAAa	24-34	aaTGTGTGAAa	95.00
V$HMGIY_Q	+	27-41	tgtgaAATTTctttt	28-42	tgtgaAATTTcctta	95.00
V$TEF1_Q6	-	84-89	CATTCC	84-89	CATTCC	85.00
V$ELK1_02	+	154-167	tcagaAGGAAgcca	154-167	tcagcGGGAAgcca	100.00
V$AFP1_Q6		167-177	ATGCAGATAAT	167-177	ATGCAGATAAT	
V$PAX8_B	+	171-188	agataattGCCTGaattt	171-188	agataattGCCTGaattt	100.00
V$NKX25_02	+	172-179	GATAATTg	172-179	GATAATTg	100.00
V$RORA1_01	+	211-223	gaatcaAAGTCAc	210-222	gaatcaAAGTCAc	100.00
V$LEF1_Q2_	+	212-221	aatCAAAGtc	211-220	aatCAAAGtc	100.00
V$LEF1B_01	-	213-219	aTCAAAG	212-218	aTCAAAG	100.00
V$TCF4_Q5	-	213-220	aTCAAAGt	212-219	aTCAAAGt	100.00
V$LEF1_Q2	+	214-219	TCAAAG	213-218	TCAAAG	100.00
V$CMYB_01	+	220-237	tcacacTGCAGTTGCtga	219-236	tcacacTGCAGTTGTtga	100.00
V$MYB_Q5_	-	225-233	ctgcaGTTg	224-232	ctgcaGTTg	100.00
V$VMYB_01	-	226-235	tgCAGTTgct	225-234	tgCAGTTgtt	100.00
V$PAX6_Q2	-	247-260	tGGTTCCAGCCCAT	246-259	cAGTTCCAGATCAT	85.00
V$NANOG_0	+	253-264	caGCCCATTGAC	252-263	caGATCATTGAC	90.00
V$HNF6_Q6	-	257-268	ccATTGACTTtt	256-267	tcATTGACTTtg	95.00
V$CEBP_C	+	261-278	tGACTTTTGCAATTTTct	260-277	tGACTTTGGCAATTTTc	95.00
V$CEBPA_01	-	262-275	gacttttgcAAttt	261-274	gacTTTGGCAAt	95.00
V$CEBP_Q2_	-	262-273	gacTTTTGCAAt	261-272	gacTTTGGCAAt	95.00
V$CEBP_Q3	+	262-273	gacttttGCAAt	261-272	gactttgGCAAt	95.00
V$HMGIY_Q	+	266-280	tttgcAATTTtcttt	265-279	ttggcAATTTtcttt	95.00

Supplementary Table 2: *In silico* identification of conserved TFs binding sites conserved in *Homo sapiens* and *Xenopus tropicalis* CNS1 using R-vista 2.0. In grey, TFs binding sites conserved between *Homo sapiens* and *Xenopus tropicalis* but not present in *Mus musculus*

Conserved TF binding site	strand	Location (Homo sapiens)	Sequence (Homo sapiens)	Location (Xenopus tropicalis)	Sequence (Xenopus tropicalis)	Matrix score
V$HMGIY_Q	-	27-41	aaagaAAATTgcaaa	265-279	ttggcAATTTtcttt	95.00
V$CEBP_C	-	29-46	agAAAATTGCAAAAGT	260-277	tGACTTTGGCAATTTT	95.00
V$CEBPA_01	+	32-45	aaaTTgcaaaagtc	261-274	gactttggcAAttt	95.00
V$CEBP_Q3	_	34-45	aTTGCaaaagtc	261-272	gactttgGCAAt	95.00
V$CEBP_Q2	+	34-45	aTTGCAAAAgtc	261-272	gacTTTGGCAAt	95.00
V$HNF6_Q6	+	39-50	aaAAGTCAATgg	256-267	tcATTGACTTtg	95.00
V$FXR_IR1_	+	41-53	AAGTCAATGGCCt	253-265	aGATCATTGACTT	90.00
V$NANOG_0	-	43-54	GTCAATGGCCtg	252-263	caGATCATTGAC	90.00
V$GCNF_01	-	46-63	aaTGGCCTGGAACTA	243-260	ttCCAGTTCCAGATCAt	90.00
V$PAX6_Q2	+	47-60	ATGGCCTGGAACTa	246-259	cAGTTCCAGATCAT	90.00
V$CMYB_01	-	70-87	tcaGCAACTGCAgtgtg	219-236	tcacacTGCAGTTGTtg	100.00
V$VMYB_01	+	72-81	agcAACTGca	225-234	tgCAGTTgtt	100.00
V$MYB_Q5_	+	74-82	cAACtgcag	224-232	ctgcaGTTg	100.00
V$RORA1_0	-	84-96	gTGACTTtgattc	210-222	gaatcaAAGTCAc	100.00
V$LEF1_Q2_	-	86-95	gaCTTTGatt	211-220	aatCAAAGtc	100.00
V$TCF4_Q5	+	87-94	aCTTTGAt	212-219	aTCAAAGt	100.00
V$LEF1_Q2	-	88-93	CTTTGA	213-218	TCAAAG	100.00
V$LEF1B_01	+	88-94	CTTTGat	212-218	atCAAAG	100.00
V$STAT3_02	+	91-98	tgaTTCCc	208-215	gGGAAtca	100.00
V$GATA1_0	-	95-107	tcccCTATCataa	199-211	gtatGATAGggga	100.00
V$GATA3_0	-	97-105	ccctATCat	208-215	gGGAAtca	100.00
V$LMO2CO	-	97-105	cccTATCat	201-209	atGATAggg	100.00
V$PAX8_B	-	119-136	aaattCAGGCaattatct	171-188	agataattGCCTGaattt	100.00
V$NKX25_0	-	128-135	cAATTATC	172-179	GATAATTg	100.00
V$AFP1_Q6	+	130-140	ATTATCTGCAT	167-177	ATGCAGATAAT	100.00
V$ELK1_02	-	140-153	tggcTTCCTtctga	154-167	tcagcGGGAAgcca	100.00
V$CMAF_01	-	175-193	ttaatgcaTCAGCAcaga	114-132	ctttgTGCTGTtgcattaa	95.00
V$TEF1_Q6	+	218-223	GGAATG	84-89	CATTCC	85.00
V$HMGIY_Q	-	266-280	aaaagAAATTtcaca	28-42	tgtgaAATTTcctta	95.00
V$RBPJK_01	-	274-284	tTTCACACAtt	24-34	aaTGTGTGAAa	95.00
V$TBX5_Q5	+	275-284	tTCACACATt	24-33	aATGTGTGAa	95.00
